# Epidemiology and palliative care of in-patient cerebral metastases cases in Germany

**DOI:** 10.1007/s11060-024-04928-4

**Published:** 2025-04-11

**Authors:** Larissa Fink, Christiane von Saß, Heidrun Golla, Raymond Voltz, Felix Muehlensiepen, Christoph J. Ploner, Philipp J. Slotty, Birgitt van Oorschot, Alexander B. Kowski, Marcel A. Kamp

**Affiliations:** 1 Brandenburg Medical School Theodor Fontane and Faculty of Health Sciences Brandenburg, Am Seebad 82/83, 15562 Rüdersdorf bei Berlin, Germany; 2 Department of Palliative Medicine, Faculty of Medicine and University Hospital, University of Cologne, Cologne, Germany; 3 Department of Palliative Medicine, University Medical Center Göttingen, Göttingen, Germany; 4 Center for Health Service Research Brandenburg, Brandenburg Medical School Theodor Fontane, Rüdersdorf, Germany; 5 Department of Neurology, Charité-Universitätsmedizin Berlin, Berlin, Germany; 6 Medical Faculty, University Hospital Düsseldorf, Heinrich-Heine-University, Düsseldorf, Germany; 7 Department of Palliative and Neuropalliative Care, Immanuel Clinic Rüdersdorf, University Hospital of the Brandenburg Medical School Theodor Fontane, Rüdersdorf bei Berlin, Germany; 8 Rangsdorf, Germany

**Keywords:** Metastatic brain tumor, Cerebral metastases, Cerebral neoplasms, Palliative care, Early integration, Mortality, Age

## Abstract

**Introduction:**

Cerebral metastases (CM) are the most common intracranial neoplasms, significantly impacting patient quality-of-life. Despite advancements in diagnostics and therapeutics, the burden remains high. This study evaluates inpatient management, palliative care use, and mortality outcomes for CM patients in German hospitals in 2022.

**Methods:**

A cross-sectional analysis was conducted on 71,787 inpatient cases involving adult CM and leptomeningeal malignancies patients in German hospitals in 2022. Data submitted by hospitals according to §21 of the Hospital Remuneration Act were analyzed, focusing on demographic data, primary tumor types, treatment methods, participation in palliative care, and discharge outcomes.

**Results:**

Among the 71,787 cases, 53.4% were patients aged 65 years or older. Malignant lung tumors were present in 61.6% of cases, followed by breast malignancies (12%) and malignant melanoma and diffuse diffuse large B-cell lymphoma (each 6.4%). Specialized inpatient palliative care (SIPC) was provided in 14.8% (10,636 cases), with 85.2% not receiving such care. Hospital mortality was 13.1% (9413 cases), with 42.2% of these involving patients who received SIPC. Discharge outcomes included discharge home (72.7%), transfers to other hospitals (7.1%), rehabilitation facilities (0.4%), nursing facilities (2%), and hospices (2.4%).

**Conclusion:**

Despite treatment advances, high mortality rates for CM patients persist, underscoring the need for palliative care integration and comprehensive training to enhance patient outcomes. Health care planning is a growing topic, our study establishes a benchmark for CM care in German hospitals, revealing a significant number of patients not receiving SIPC. This research can inform future healthcare strategies in neuro-oncology.

**Supplementary Information:**

The online version contains supplementary material available at 10.1007/s11060-024-04928-4.

## Introduction

Palliative care aims to alleviate distressing symptoms and improve the quality-of-life for patients with potentially life-threatening diseases. The diagnosis of cerebral metastasis (CM) presents significant physical, psychological, social, and spiritual challenges, greatly diminishing quality-of-life (QoL) [1–3].

Several factors contribute to the increasing burden of CM: The incidence of CM appears to be rising in the United States (U.S.) [4–6]. This increase is challenging to quantify due to the lack of comprehensive national registries in Germany and other European countries, such as the Central Brain Tumor Registry of the U.S. [5, 7]. However, improved diagnostic methods and their routine use have led to higher detection rates. Currently, estimates suggest up to 400,000 new cases of CM are diagnosed annually in the United States, with 10–40% of patients with solid tumors developing CM during their illness [7]. Advancements in primary tumor treatments have extended patient survival, resulting in more frequent detection of CM over the disease course. Additionally, overall survival of CM patients may increase: Early landmark studies reported a median survival time of up to 9 month for patients with a single CM receiving surgery with whole-brain radiation therapy (WBRT) compared to WBRT alone [8]. More recent studies, such as the EORTC 22952-26001, noted median survival times of 10.9 to 10.7 months for patients with 1–3 CMs treated with surgery or stereotactic radiosurgery (SRS) with or without WBRT [9]. The NCCTG N107C/CEC 3 study showed median survival times of 12.2 and 11.6 months for single resected metastases treated with postoperative SRS versus WBRT [10]. Studies using the diagnosis-specific Graded Prognostic Assessment (ds-GPA) indicate even better prognoses for certain subgroups, with median survival up to 46 months [11]. Despite these advancements, most patients with CM remain incurable.

The increasing incidence of cerebral metastases poses a challenge for the healthcare system. However, it remains unclear whether this rise, coupled with improved survival times, translates to a higher individual burden for patients. CM can cause a wide range of distressing symptoms, including physical symptoms such as intracranial pressure, headaches, focal neurological deficits, and epilepsy. They also lead to psycho-oncological challenges like anxiety and distress, social burden such as limited participation and care needs, and spiritual concerns [3, 12–14]. Palliative care aims to alleviate these symptoms, achieve symptom control, and enhance QoL [15]. In Germany, specialized in-patient palliative care (SIPC) is designed to address the needs of hospitalized patients with complex symptoms, delivered by multidisciplinary teams with expertise in palliative care.

The frequency of SIPC for CM patients in German hospitals is unclear, and data on CM hospital cases and SIPC involvement are lacking. This study aims to determine the prevalence of CM cases, the frequency of SIPC use in comparison to other metastatic tumors other than CM, and the inpatient mortality rates for CM patients in German hospitals.

## Methods

### Ethics approval and data availability

This study adhered to the ethical principles outlined in the 1964 Helsinki Declaration and its subsequent amendments. The institutional and local ethics committee (Study ID: 190032024-ANF, Brandenburg Medical School, Germany) approved the study protocol. The results conform to the STROBE guidelines for reporting observational studies (Supplementary document 1) [16]

### Study design, setting and data source

In this cross-sectional study, we analyzed aggregated data from all German hospital cases for the year 2022. The data were obtained from the Institute for the Remuneration System in the Hospital Sector (InEK GmbH, Siegburg, Germany).

### Cohort/participants and study size

We identified the cohort of hospital cases diagnosed with CM using the following inclusion criteria: (1) diagnosis of CM and/or leptomeningeal malignancies, identified by the International Statistical Classification of Diseases and Related Health Problems (ICD)-10-German modification (ICD-10-GM) code C79.3, (2) treated in 2022 and (3) patients aged 18 and above. Due to technical constraints, our analysis focuses on hospital cases rather than individual patients. The study size reflects the number of cases treated in 2022. For comparison, we identified and analyzed comparable cohorts of hospitalized cases with lung, bone, adrenal, and liver metastases.

### Definitions and variables

Diagnoses were defined using ICD-10-GM codes, and medical diagnoses and treatments were specified by their procedural codes (*Operationen- und Prozedurenschlüssel*, OPS) [17, 18]. The definitions of the relevant OPS codes are detailed in Supplementary document 2. The list of comorbidities is based on the Charlson Comorbidity Index [19].

In addition to diagnoses and procedures, the following variables were determined:

- The proportion of hospital cases involving patients with CM and/or leptomeningeal malignancies.
- Total number of hospital cases for each analyzed cohort.
- Distribution of sex and age.
- Distribution of treating hospitals, categorized by bed size and hospital provider.
- Reasons for hospital discharges.

### Bias

We minimized selection bias by including all 2022 German hospital cases with CM, ensuring a comprehensive sample. Our analysis includes all cases billed in the DRG system, except for cases fewer than five, excluded for data protection reasons. The number of exclusions for data protection reasons should not affect the overall outcome. About 70 palliative care units operating outside the DRG system are not included. The study design permits only the analysis of hospital cases, not individual patients. To reduce measurement bias, we extracted data on palliative care and intensive care treatments using predefined OPS and ICD-10 codes, ensuring consistent identification of all cases receiving SIPC. While some misclassifications may arise, measurement errors are minimal based on billing data. Undoubtedly, relevant data—such as the timing and sequence of therapies, integration of SIPC, and patients' general condition, QoL and health status—are unavailable due to the study design and reliance on billing data. We sought to account for patients' disease status by listing relevant comorbidities and conducting a separate analysis of the cohort of patients who died in the hospital.

### Data handling and statistics

Data were retrieved from the InEK data browser and organized using Microsoft Excel for Mac (Version 16.78, Microsoft Corporation, Redmond, Washington, USA). Statistical analyses and graphing were conducted using GraphPad Prism 9 for macOS (Version 9.5.0, GraphPad Software, Inc., La Jolla, USA).

Descriptive statistics were employed and frequencies and ratios calculated. To calculate the number of cases for a particular diagnosis, we retrieved counts of hospital cases where the diagnosis was listed as either primary or secondary. We then subtracted double-coded cases to obtain the final count for each diagnosis. The proportion of hospital cases involving patients with CM was calculated: (Number of hospitalizations of patients with CM and a solid malignancy of one type/Total number of hospitalizations of patients diagnosed with the solid malignancy of this type) × 10^2^. The cohorts of patients with other metastases were calculated analogously.

## Results

### Baseline characteristics of the patient cohort with cerebral metastases

In 2022, German hospitals treated adult patients in 14,770,158 hospital cases, thereof patients with primary malignancies (ICD-10-GM code C00-75) in 1,643,185 cases and hematologic malignancies (C82–C96) in 212,129. In 249,561 hospital cases, patients suffered from lung malignancies, in 202,472 cases breast cancer, and in 152,087 cases from prostate malignancies.

**Fig. 1:**
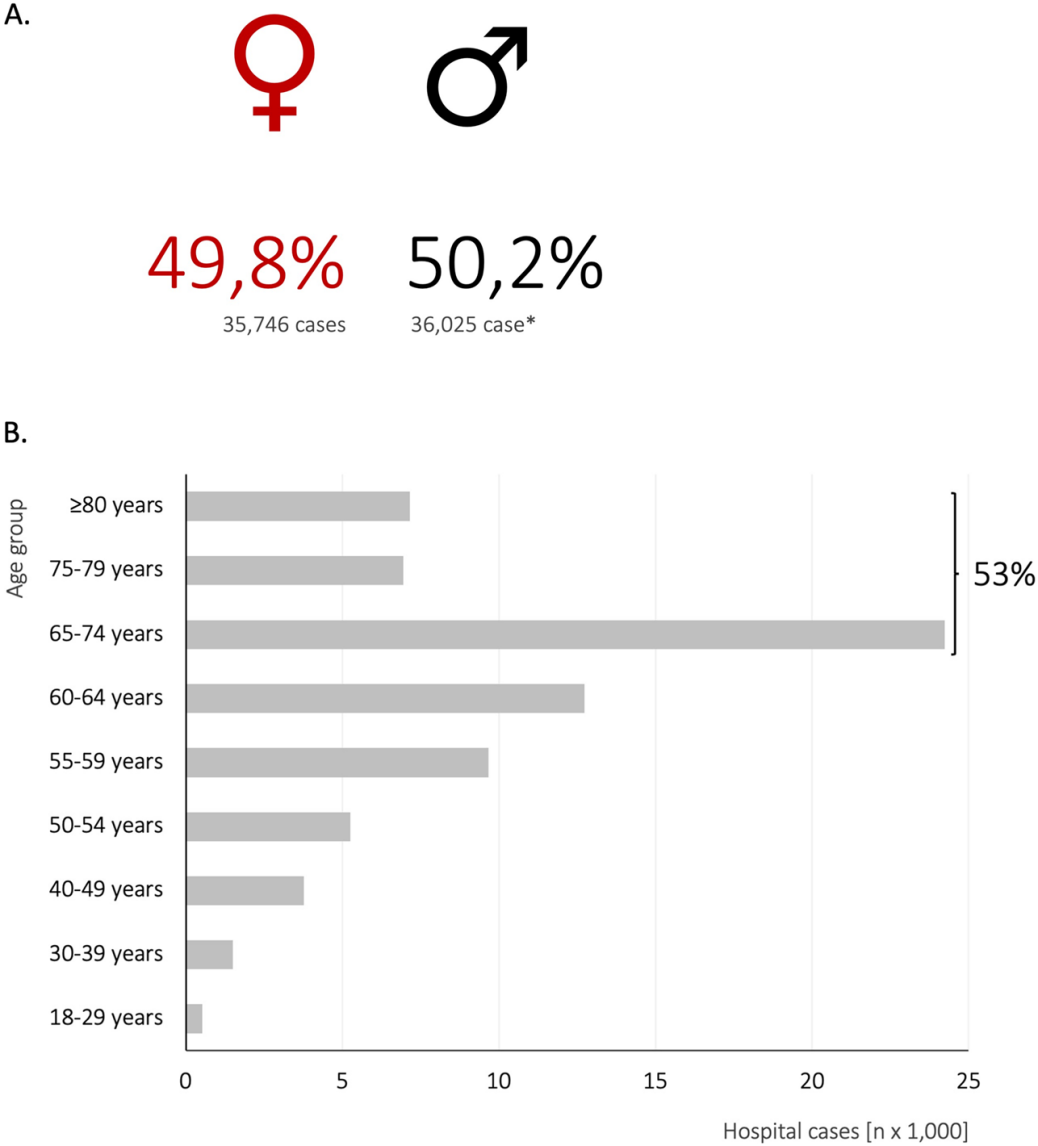
Age and gender distribution. **A** illustrates the gender distribution in hospital cases involving cerebral metastases in Germany in 2022, while **B** shows the corresponding distribution across different age groups

In the same period, German hospitals managed 71,787 inpatient cases involving adult CM-patients (0.5% of all hospital cases). Among these, 14,961 cases listed CM as the primary diagnosis and 60,428 cases as a secondary diagnosis (including 3602 cases of double coding). Female patients accounted for 36,025 cases (50.2%). Patients aged ≥ 65 years represented 38,335 cases (53.4%, Fig. 1; Table 1). In 2022, patients with liver metastases were treated in 174,308 cases, with bone metastases in 154,710 cases, with lung metastases in 120,437 cases and with adrenal metastases in 24,405 cases (Supplementary Table 1).

**Table 1:** Age groups

	All cerebral metastases cases		SIPC cases		Intensive care cases	
	Number of hopital cases	%	Number of hopital cases	%	Number of hopital cases	%
Total	71,787	100	10,636	100	10,250	100
*Sex*						
Female	35,746	48.8	5819	54.7	5389	52.6
Male	36,025	50.2	4814	45.3	4856	47.4
Diverse	1	0	0	0	1	0
Unknown	15	0	3	0	4	0
*Age groups*						
18–29 years	527	0.7	35	0.3	64	0.6
30–39 years	1506	2.1	238	2.2	250	2.4
40–49 years	3769	5.3	538	5.1	573	5.6
50–54 years	5257	7.3	759	7.1	772	7.5
55–59 years	9654	13.4	1277	12.0	1431	14.0
60–64 years	12,739	17.7	1762	16.6	1735	16.9
65–74 years	24,249	33.8	3622	34.1	3388	33.1
75–79 years	6936	9.7	1115	10.5	984	9.6
≥ 80 years	7150	10.0	1290	12.1	1053	10.3
≥ 65 years	38,335	53.4	6027	56.7	5425	51.0

### Primary tumors and metastasis distribution

**Fig. 2:**
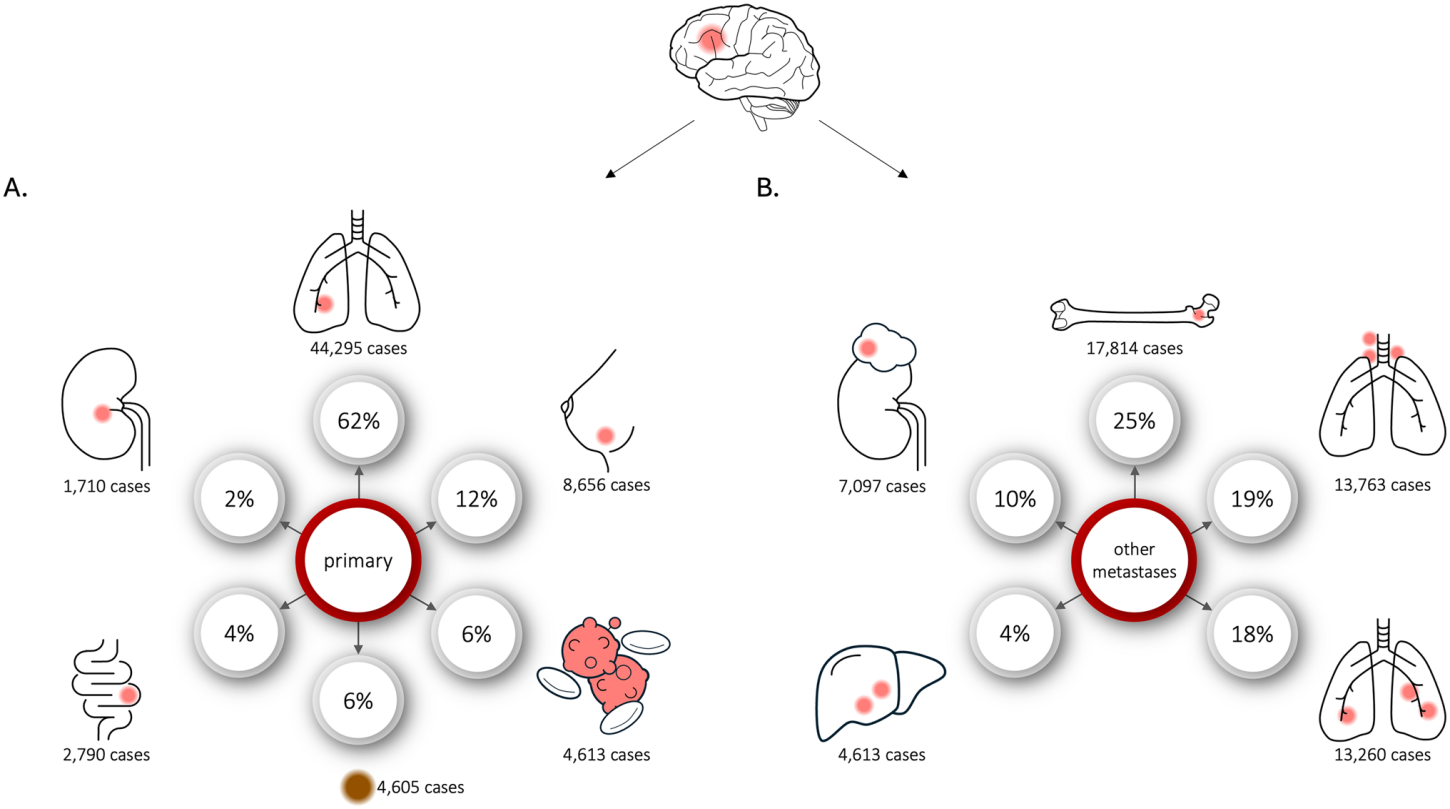
Most common primary tumors and metastatic spread in hospital cases involving cerebral metastases patients

**Fig. 3:**
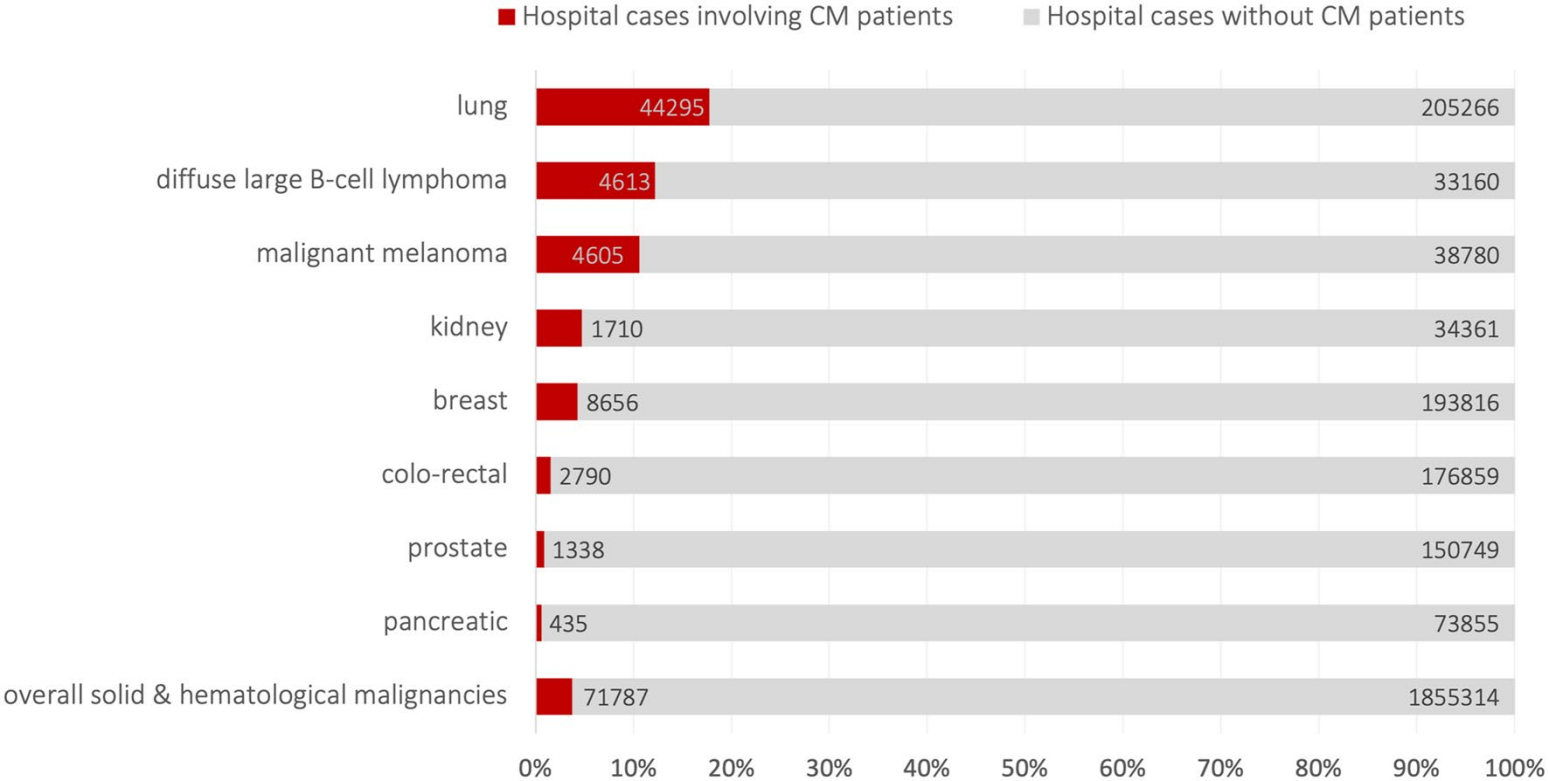
Proportion of hospital cases involving patients with cerebral metastases, relative to the total number of hospitalizations for each type of malignancy. The number within the bars indicate the case number for hospital cases with cerebral metastases patients (red bar) and patients without cerebral metastases (grey bar)

In 44,295/71,787 cases, CM-patients had malignant lung tumors (61.6%), in 8656 cases breast malignancies (12%) and in 4605 cases malignant melanoma (6.4%; Fig. 2A; Table 2). Additionally, 4613 cases (6.4%) involved diffuse large B-cell lymphoma and 2789 cases (3.9%) involved malignant neoplasms of unknown primary locations. The proportion of hospital cases involving patients with CM, relative to the total number of hospitalizations for each type of malignancy, was 17% for lung malignancies (44,295/249,561), 4.2% for breast malignancies (8656/202,472), 10.6% for malignant melanoma (4605/43,385) and 12.2% for diffuse large B-cell lymphoma (4613/37,773 cases, Fig. 3; Table 2). CM patients suffered from bone or bone marrow metastases in 17,814 cases (24.8%), intrathoracic lymph node metastases in 13,763 cases (19.5%), lung metastases in 13,260 cases (18.4%, Fig. 2B; Table 2). Patients with cerebral metastases had a range of significant comorbidities. Among the 71,787 cases, there were 13,112 codes for ulcers (18.3%), 10,881 codes for diabetes mellitus (15.2%), 9371 codes for chronic pulmonary disease (13.1%), and 8798 codes for renal failure (12.3%). A detailed list of relevant comorbidities, based on the Charlson Comorbidity Index, is provided in Supplementary Table 2.

**Table 2:** Primary and metastatic cancer in hospital cases involving patients with cerebral metastases

Organ	ICD-10-GM code	Total number of hospital cases	Hospital cases with patients suffering from cerebral metastases	%
	*Primary cancer*			
Overall	**C00-75**	**1,643,185**	**71,787**	**4.4**
Colo-rectal malignoma	C18-C20	179,649	2790	1.6
Pancreatic malignoma	C25	74,290	435	0.6
Lung malignoma	C34	249,561	44,295	17.7
Malignant melanoma	C43	43,385	4605	10.6
Breast malignoma	C50	202,472	8656	4.3
Prostate malignoma	C61	152,087	1338	0.9
Kidney malignoma	C64	36,071	1710	4.7
Primary location unknown	C80.0	32,808	2789	8.5
	*Metastatic cancer in hospital cases involving cerebral metastases patients*			
Lung	C78.0	120,437	13,260	11.0
Pleura	C78.2	45,146	4053	9.0
Liver & intrahepatic bile ducts	C78.7	174,308	12,588	7.2
Retroperitoneum & peritoneum	C78.6	82,924	2040	2.5
Other & unspecified digestive organs	C78.8	9256	1462	15.8
Kidney & renal pelvis	C79.0	4400	1249	28.4
Skin	C79.2	12,479	1326	10.6
Bone & bone marrow	C79.5	154,710	17,814	11.5
Adrenal gland	C79.7	24,405	7097	29.1

The bold line give the total number of cases, which is the total number of hospital cases coded with ICD-10 code C00-C75 and the number of cases suffered from CM

### Antitumor therapies and utilization of specialized inpatient palliative care

Public hospitals with over 1000 beds treated most cases (16,798 cases; 23,4%), followed by public hospitals with 800 to 999 beds (4966 cases, 6.9%). Oncology departments were the primary treating discipline in 18,905 cases (26.3%), neurosurgery clinics in 8060 cases (11.2%), neurology departments in 7522 cases (10.5%), radiotherapy in 6148 cases (8.6%), and designate palliative care clinics in 3695 cases (5.1%).

Neurosurgical excision of CM was conducted in 5673 cases (7.9%). Radiotherapy was administered under inpatient conditions in 15,307 cases (21.3%), chemotherapy in 17,940 cases (25%), and immunotherapy in 15,432 cases (21.5%, see Table 3). An ICU treatment was performed in 10,250 cases (14.3%).

Among the 71,787 hospital cases involving patients with CM, 10,636 cases (14.8%) received 11,011 different SIPC procedures (Fig. 3, for age and sex distribution see Table 1). This included 3562 cases of complex palliative care (OPS code 8–982), 5172 cases on a palliative care ward (OPS code 8-98e), and 2277 cases by a palliative care consultation service (OPS code 8-98h). In the majority of CM hospital cases with SIPC, cerebral metastases were coded as secondary diagnosis in the majority of cases, and as the primary diagnosis in CM hospital cases with SIPC in which patients died (Supplementary Table 3). SIPC was provided in 6844 of 44,295 cases (15.5%) of lung malignancies, 1846 of 8656 cases (21.3%) of breast malignancies, 623 of 4605 cases (13.5%) of malignant melanomas and 226 of 4613 cases (4,9%) with diffuse large B-cell lymphoma. For comparison, 23,019 SIPC procedures were conducted in 154,710 cases (14.9%) of bone metastases patients and 16,272 SIPC procedures in 120,437 cases (13.5%) of lung metastases patients (Supplementary Table 4).

### Reasons for discharge, mortality and palliative care utilization for dying patients

**Fig. 4:**
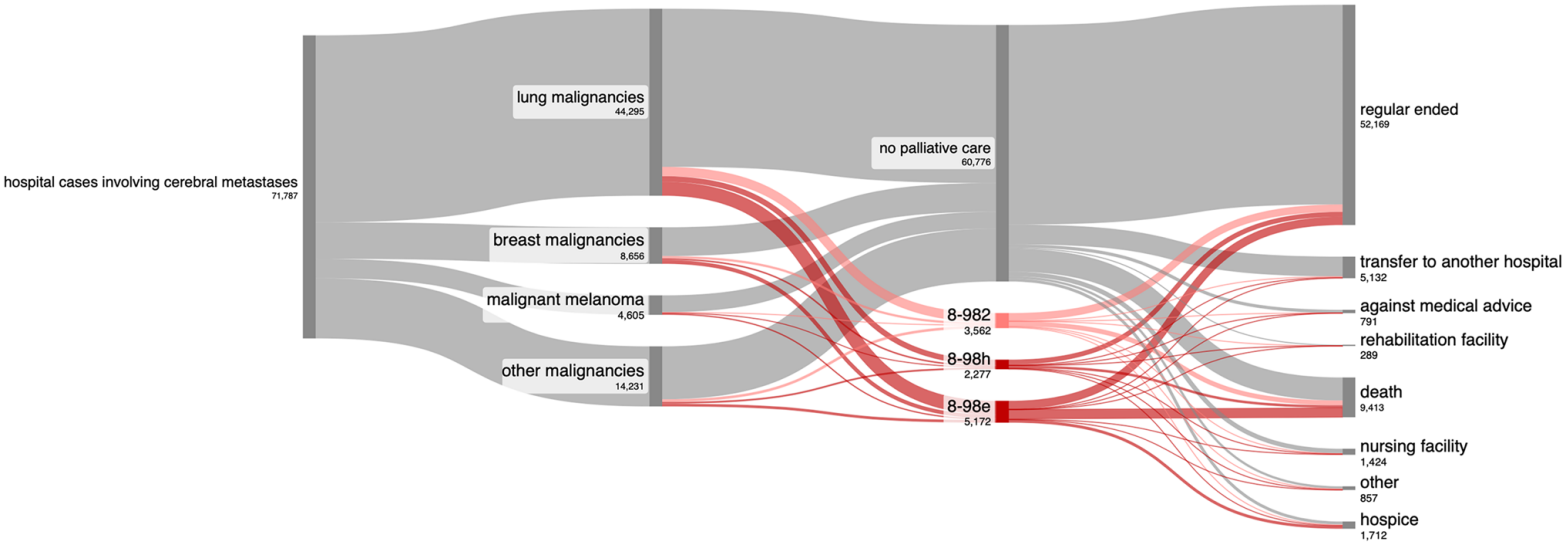
Utilization of specialized palliative care. The Sankey diagram illustrates the utilization of specialized palliative care in hospital cases in patients suffering from cerebral metastases. (legend: 8-982: complex palliative care; 8-98e: specialized palliative care on a palliative care ward; 8-98h: specialized palliative care by a palliative care consultation service)

The majority of hospital stays (52,169 cases, 72.7%) concluded with a discharge home. In 5132 cases (7.1%), patient were transferred to other hospitals, in 289 cases (0.4%) to rehabilitation facilities, in 1424 cases (2%) to nursing facilities, and in 1712 cases (2.4%) to hospices (Fig. 4).

**Fig. 5:**
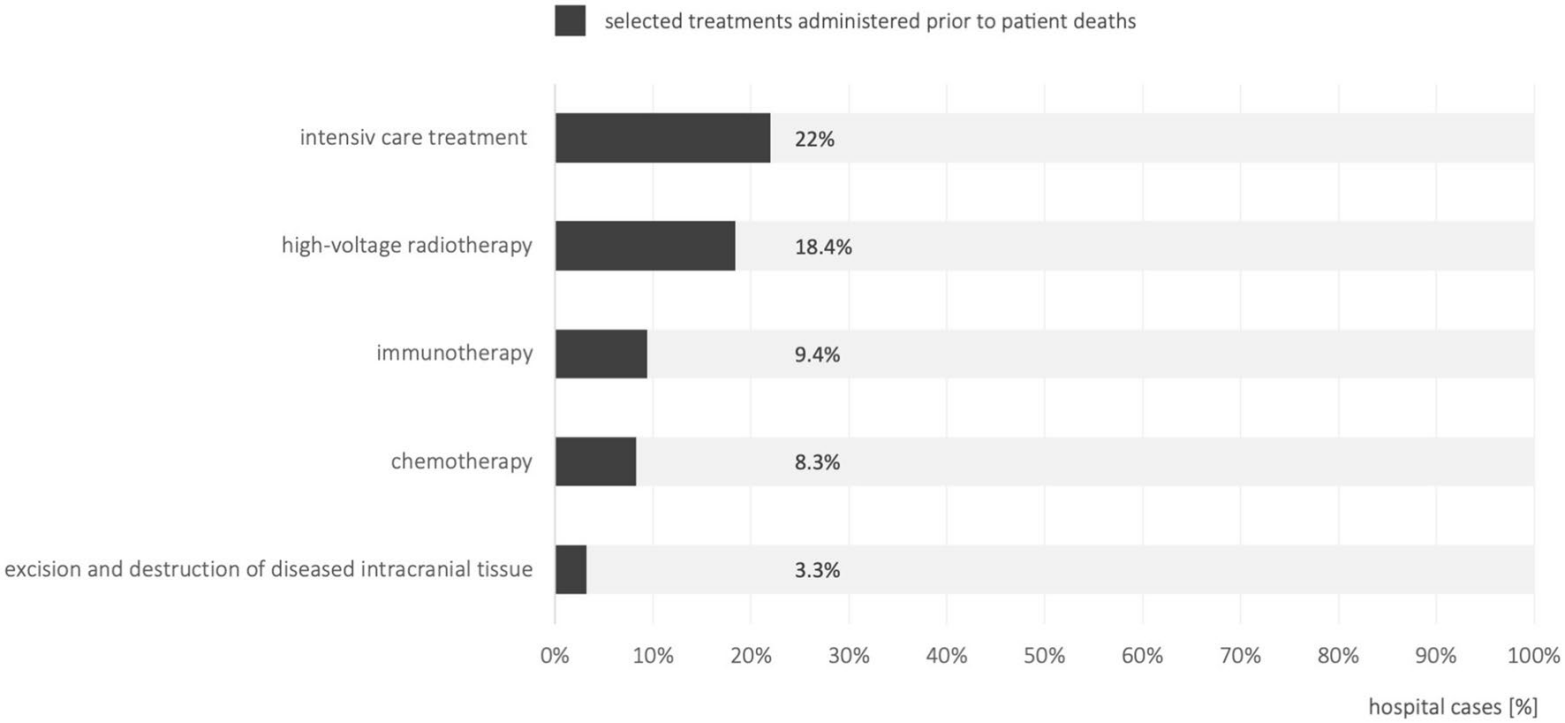
Antitumor treatments administered during hospitalization in the final stages prior to patient death

9413 patients died during their hospital stay, corresponding to a mortality rate of 13.1%. Among these, 303 (3.2%) patients underwent tumor resection for cerebral space-occupying lesions during their inpatient stay. Chemotherapy was administered to 8.3% of patients (783 patients), immunotherapy to 9.4% (889 patients) and radiotherapy to 8.4% (1735). Intensive care unit (ICU) stays were recorded in 22% of patients (2071 patients, Fig. 5) during their final hospital admission. Among the deceased patients, SIPC procedures were conducted in 3971 cases (42.2%; Fig. 4), thereof SIPC on a palliative care ward in 2245 cases (23.8%). In a total of 394 cases, patients had a stay in an intensive care unit and a palliative care unit in the same inpatient stay, of which 186 patients died. For comparison, mortality rates ranged between 12.1 and 13.7% for the cohorts with metastases other than CM and SIPC treatments in deceased patients between 38.4 and 41.3%.

Of the cases with SIPC, 37% of patients died during hospitalization (3961 cases). Mortality rates varied depending on the type of palliative care provided: 26.4% for consultation services, 31.5% (1123 cases) for complex palliative care, and 43.2% (2236 cases) for care in palliative care units. Among those who received SIPC, 4876 patients (44.3%) were discharged home, 1015 (9.2%) were transferred to a hospice, and 340 (3.1%) were moved to a nursing home.

## Discussion

Our main results on German hospital cases involving CM patients:

1. Of the 71,787 inpatient cases of CM, 61.6% had malignant lung tumors, 12% had breast malignancies, and 6.4% had malignant melanoma.
2. SIPC was provided in 10,636 cases with 11,011 different SIPC procedures (14.8% and 15.3%, respectively)
3. Mortality was 13.1% (9413 cases), with 42.2% (3971 cases) of these patients receiving SIPC.

While several studies have investigated the epidemiology of CM, there is a notable lack of comprehensive data specific to Germany and Europe. Our study offers valuable "real-world" data that enhance our understanding of the epidemiology of CM. CM most commonly originate from lung, breast, and malignant melanoma cancers, with renal cell and colorectal carcinomas following in prevalence. A recent retrospective German study involving 1248 patients who underwent radiotherapy for CM found that lung carcinomas were the most common, accounting for 45% of cases, followed by malignant melanomas at 20% and breast carcinomas at 17.5% [20]. German neurosurgical cohorts report a similar high proportion of lung cancer cases, though they note lower incidences of malignant melanomas and breast carcinomas [21–24]. In 2022, in the majority of German hospital cases, CM patients had lung tumors (61.6%), followed by breast tumors (12%) and malignant melanomas (6.4%). Among hospitalized cases, 17% of lung cancer patients, 4.2% of breast cancer patients, and 10.6% of malignant melanoma patients had CM. These findings are consistent with previous studies, which reported incidence rates of approximately 20% for lung carcinoma, 5% for breast cancer, and 7–10% for malignant melanoma [1, 8, 24]. Notably, 53.4% of the patients with CM in our study were aged ≥ 65 years (Table 3).

**Table 3:** Antitumoral and specialized palliative care treatments in hospital cases involving patients with cerebral metastases

			All patients		Deceased patients		
			n	%	n	%	% (deceased/all pts.)
*Total number of hospital cases*			**71,787**		**9413**		**13.1**
1-510.0		Biopsy of intracranial tissue	261	0.4	21	0.2	8.0
1-511		Stereotactic biopsy of intracranial tissue	358	0.5	28	0.3	7.8
5-015		Excision and destruction of diseased intracranial tissue	5673	7.9	303	3.2	5.3
8-522—8-523		High-voltage radiotherapy	15,239	21.2	1735	18.4	11.4
8-542—8-544		Non-complex chemotherapy	17,940	25.0	783	8.3	4.4
		Moderately complex and intensive block chemotherapy					
		Highly complex and intensive block chemotherapy					
8-547		Immunotherapy	15,432	21.5	889	9.4	5.8
Intensive care treatment			10,250	14.3	2071	22.0	20.2
*Specialized palliative care*			**19,745**	**27.5**	**3971**	**42.2**	**42.2**
8-982		Complex inpatient palliative care	3562	5.0	1135	12.1	12.1
	8-982.0	≤ 6 treatment days	768	1.1	382	4.1	
	8-982.1	7–14 treatment days	1627	2.3	444	4.7	
	8-982.2	14–20 treatment days	723	1.0	178	1.9	
	8-982.3	≥ 21 treatment days	444	0.6	131	1.4	
8-98e		Specialized inpatient palliative care on a palliative care ward	5172	7.2	2245	23.8	23.8
	8-98e.0	≤ 6 treatment days	1199	1.7	875	9.3	
	8-98e.1	7–14 treatment days	2126	3.0	775	8.2	
	8-98e.2	14–20 treatment days	1080	1.5	351	3.7	
	8-98e.3	≥ 21 treatment days	767	1.1	244	2.6	
8-98h		Specialized inpatient palliative care by a consultation service	2277	3.2	591	6.3	6.3
	8-98h.00	≤ 2 h	348	0.5	95	1.0	
	8-98h.01	2–4 h	620	0.9	171	1.8	
	8-98h.02	4–6 h	480	0.7	118	1.3	
	8-98h.03	6–9 h	386	0.5	115	1.2	
	8-98h.04	9–12 h	207	0.3	50	0.5	
	8-98h.05	12–15 h	106	0.1	23	0.2	
	8-98h.06	15–20 h	81	0.1	13	0.1	
	8-98h.07	20–25 h	26	0.0			
	8-98h.08	25–35 h	23	0.0	6	0.1	

First bold line: total number of all hospital cases with cerebral metastases patients (all vs. deceased) and, 2nd bold line, all cases with CM pts. received palliative care within the DRG system.

Therapy recommendations for CM and leptomeningeal carcinosis patients are well-established in various guidelines, such as those from ASCO-SNO-ASTRO or EANO-ESMO [25–29]. Surgical therapy is a standard approach, particularly for patients with oligometastatic, symptomatic, large, and superficial metastases. Stereotactic radiation therapy is commonly employed for treating individual, smaller, deeper metastases. Whole-brain radiation therapy, often with hippocampal sparing, is indicated for cases with extensive multiple metastases. Treatment of leptomeningeal malignancies typically involves of chemotherapeutic and radiotherapeutic concepts [26, 27, 29]. Additionally, guideline weakly recommend certain chemo-/immuno- and targeted therapies. Despite these clear recommendations, there is little information on how often the individual therapy modalities are used in real life. In 2022, tumor resections were performed in 8% of hospital cases involving patients with CM and leptomeningeal malignancies, chemotherapy and immunotherapy in 25%, and 21% of cases. However, it is important to note that these numbers do not reflect the overall usage of these therapies among these patients, as many may have received treatment on an outpatient basis. Additionally, it is likely that many patients were hospitalized multiple times within a year, while surgeries and other therapies were performed during only one or a few of these hospital stays. Consequently, the proportion of patients who underwent surgery or received other specific therapies may be higher.

The prognosis for CM patients varies depending on the primary tumor, genetic and molecular factors, but remains limited, with survival often ranging between 3 months and nearly 4 years [11]. Prognosis for patients with leptomeningeal metastasis is 2–6 month [26]. In our study, the inpatient mortality rate for hospital cases involving CM patients was 13.1%, comparable to mortality rates in hospital cases with bone, lung, or liver metastases.

The timely integration of palliative care and the avoidance of both over- and under-provision are key indicators of quality in oncological therapies. Although the ASCO-SNO-ASTRO and EANO-ESMO guidelines for brain metastases do not focus on palliative care, an ASCO guideline recommends early integration of SIPC within 8 weeks of diagnosis for tumors [30]. In Germany, under-provision has been defined, among other criteria, as a low rate of specialized palliative care at the end of life [31]. In 2022, the rate of SIPC treatment for CM patients in Germany was 14,8% for all hospital cases and 42.2% for cases in which CM patients died. These rates were slightly higher than those for patients with bone, lung, or liver metastases, although ideal rates remain undefined. Our results are limited by the study design, which focuses on the analysis of hospital cases rather than individual patients, excludes specialized facilities, and lacks relevant information such as quality-of-life data. Quality indicators for end-of-life care also include the number of chemotherapy treatments administered within the last 30 days before death [31, 32]. Our study could not assess the duration and timing of tumor-directed therapies before death. However, data from Germany in 2022 indicates that 8.3% of hospital cases of deceased CM patients received chemotherapy, 9.4% received immunotherapy, 18.4% received radiation therapy, and 3.2% underwent brain tumor resection during their final hospital stay. Historical data from 2010 to 2014 shows that 12.2% of 80,768 deceased cancer patients received chemotherapy in their last month of life [31]. Similarly, a recent study involving 145,372 BARMER health insurance policyholders who died between 2016 and 2019 found that 12.2% received chemotherapy in their final 30 days [32, 33]. These findings underscore the importance of integrating palliative care into neuro-oncological training and contribute to the ongoing evaluation of quality factors in oncological and palliative care [34–36].

Our study offers insights into the epidemiology and inpatient care of patients with cerebral metastases, serving as a foundation for future health care planning, as well as the organization and optimization of care for CM-patients. This encompasses both tumor therapy and palliative care. The SIPC rate of 13.1% among all CM-patients, and 42% for those nearing end of life, appears notably low, suggesting a need for improvement.

### Limitations

This study has several limitations:

1. The number of hospital cases does not directly correspond to individual patients. Individual patients may experience multiple hospitalizations within a year, but may only receive SIPC during one of those hospitalizations. As a result, the overall rate of CM patients who have a SIPC at any point during their disease course might be higher.
2. The secondary diagnosis of C79.3 might be coded multiple times within the same case, potentially lowering the actual count. Such instances are likely minimal.
3. Approximately 70 palliative care units in Germany are classified as "special facilities" (*besondere Einrichtungen*), remunerating outside the DRG system. These units handle totaling approximately 17,500 cases across all palliative care entities [37], but cannot be integrated in our dataset.
4. Hospital mortality was calculated based on hospital cases, but specific causes of death were not specified. Nevertheless, hospital mortality remains a relevant parameter for patient advice.
5. We lack data on the outpatient care of neuro-oncological patients, which is relevant for chemotherapy and radiotherapy, as most treatments are outpatient.
6. The findings' reliability depends on accurate and consistent coding practices in the InEK database. Misclassification or inconsistent coding could introduce biases or inaccuracies. However, prior research has reported high reliability within the German healthcare context [38]. Additionally, data on the quality-of-life are crucial but unavailable in this study.
7. The time sequence of anti-tumor therapies, intensive care, and SIPC (whether synchronous or sequential) is not available.
8. Data provided do not cover key aspects of palliative care, such as symptom burden, quality-of-life, and stress experienced by CM patients and their families. Thus, the actual assessment of palliative care demand is impossible. Not all ICU patients require SIPC, as basic palliative care might have been provided by other physicians.

## Conclusion

In 2022, German hospitals managed 71,787 inpatient cases involving CM patients and/or patients with leptomeningeal cancer. SIPC was provided in 14.8% of cases, and 13.1% of patients died, with 42.2% receiving SIPC. Despite the limitations of this analysis, these findings describe the status of SIPC treatments for CM patients in Germany. Future efforts may focus on enhancing the integration of palliative care into neuro-oncological hospital practice and establishing reference values for the extent of specialized palliative care. Future efforts should prioritize the better incorporation of palliative care into neuro-oncological hospital practice. Our study offers key data on CM epidemiology and inpatient care, providing a foundation for optimizing both tumor therapy and palliative care in health planning. The current SIPC rates suggest significant room for improvement.

## Supplementary Information

Below is the link to the electronic supplementary material.

Supplementary file1 (DOCX 32 KB)

Supplementary file2 (DOCX 23 KB)

Supplementary file3 (XLSX 17 KB)

Supplementary file4 (XLSX 22 KB)

Supplementary file5 (XLSX 15 KB)

Supplementary file6 (XLSX 11 KB)

## Data Availability

No datasets were generated or analysed during the current study.
