# Supplementary material for: Epidemiology and palliative care of in-patient cerebral metastases cases in Germany
Source: J Neurooncol. 2025 Apr 11;173(1):37–48. doi: 10.1007/s11060-024-04928-4 (PMC12041120; doi:10.1007/s11060-024-04928-4)
Supplement: Supplementary file 2 — Supplementary file2 (DOCX 23 KB) [file 11060_2024_4928_MOESM2_ESM.docx]

supplemental document 2:

Definition of palliative care OPS-Codes

**8-982 Complex palliative care**

*Not included:*

- Specialized inpatient complex palliative care treatment (8-98e)
- Specialized complex palliative care treatment by a palliative care service (8-98h)

*Required structural features:*

- Treatment led by a specialist with additional qualification in palliative medicine

*Minimum features:*

- Conducting a standardized palliative medicine basic assessment (PBA) at the start of treatment
- Holistic treatment for symptom control and psychosocial stabilization of patients with a progressive, advanced illness and limited life expectancy, if necessary, with the involvement of their relatives
- Creation and documentation of an individual treatment plan upon admission
- Patient-specific documentation of palliative medicine treatment goals and treatment results
- Weekly multi-professional team meeting with the presence of the medical treatment manager and at least one member of the nursing team and at least one other representative of the professional groups involved in patient care per full week
- Use of at least 2 of the following therapy areas: social work/social education, psychology, special education, physiotherapy/occupational therapy, artistic therapy (art and/or music therapy), relaxation therapy and conducting patient, relative and/or family discussions with a total of at least 6 hours per patient and full week patient-related in different combinations (the patient, relative and/or family discussions can be attended by all professional groups of the treatment team.) If two or more representatives of different professional groups of the treatment team are used simultaneously, the respective employee minutes are added up

**8-98e Specialized inpatient palliative medical complex treatment** *Not included:*

- Complex palliative medical treatment (8-982)
- Specialized complex palliative medical treatment by a palliative care service (8-98h)

*Required structural features:*

- Existence of an independent palliative care unit (at least 5 beds) with a multi-professional team specializing in particularly complex and demanding palliative care
- Professional treatment management by a specialist with additional qualifications in palliative medicine and at least 6 months of experience in treating palliative patients in a palliative care ward or in another specialized palliative care facility. 24-hour professional treatment management can be ensured by on-call
- At least 7 hours of medical presence in the palliative care unit on working days
- Nursing management with proof of a recognized curricular additional qualification in palliative care of at least 160 hours and with at least 6 months of experience in a specialized palliative care facility
- Existence of specialized device-based palliative medical treatment procedures with the possibility of continuous monitoring, e.g., pain pumps and other continuous parenteral therapies for symptom control

*Minimum features:*

- Conducting a standardized palliative medicine basic assessment (PBA) at the start of treatment
- Daily multi-professional case discussion with attendance documentation
- Creation and documentation of an individual treatment plan upon admission
- Patient-specific documentation of palliative medicine treatment goals and treatment results
- Holistic treatment for symptom control and psychosocial stabilization of patients with a progressive, advanced illness and limited life expectancy, if necessary, with the involvement of their relatives
- Weekly multi-professional team meeting with the presence of the medical treatment manager and at least one member of the nursing team and at least one other representative of the professional groups involved in patient care per full week
- Use of at least 2 of the following therapy areas: social work/social education, special education, psychology, physiotherapy/occupational therapy, artistic therapy (art and/or music therapy), relaxation therapy and conducting patient, relative and/or family discussions with a total of at least 6 hours per patient and full week, patient-related in different combinations (The Patient, relative and/or family discussions can be carried out by all professional groups of the treatment team.) If two or more representatives of different professional groups of the treatment team are deployed simultaneously, the respective employee minutes are added up
- If necessary, referral to qualified and continuous support offers for relatives (also after the death of the patient)
- Referral and transfer to subsequent forms of general and specialized palliative care with particular consideration of emergency planning, structured instructions for relatives, social legal advice and referral, if necessary

**8-98h Specialized complex palliative care treatment by a palliative care service**

*Not included:*

- Complex palliative care treatment (8-982)
- Specialized inpatient complex palliative care treatment (8-98e ff.)

A code from this area must only be entered once per in-patient stay

*Required structural features:*

- A cross-departmental, organizationally independent, multi-professional team specializing in complex palliative care (palliative care service), consisting of a medical service, a nursing service and at least one representative from another area: social work/social education, psychology/psychotherapy, physiotherapy, occupational therapy. It offers its services for the co-treatment of patients in a case-leading department and coordinates these with the case-leading department
- Medical treatment management by a specialist with additional qualification in palliative medicine and nursing management by a nursing professional with proof of a recognized curricular palliative care additional qualification of at least 160 hours (each with at least 6 months experience in specialized palliative care)
- 24-hour availability and, if professionally necessary, the presence of a specialist with at least 6 months experience in specialized palliative care who is familiar with the patients' current problems. Outside of regular working hours, this specialist does not have to be part of the organizationally independent palliative care service team, but must be familiar with the patients' current problems

*Minimum features:*

- Implementation of a standardized palliative medicine basic assessment (PBA) at the start of treatment by the palliative care service
- Creation of an individual treatment plan agreed with the case-leading department at the start of treatment by the palliative care service
- Patient-specific documentation of palliative medicine treatment goals and treatment results by the palliative care service
- Active, holistic treatment for symptom control and psychosocial stabilization of patients with a progressive, advanced illness and limited life expectancy, if necessary, with the involvement of their relatives, in addition to the treatment by the case-leading department
- Weekly team meeting of the palliative care service with the presence of the medical treatment manager and at least one member of the palliative care service nursing staff as well as at least one other representative of the professional groups of the palliative care service involved in patient care per full week
- Forward care planning and coordination of palliative care, e.g., by setting indications for mediation and transfer to subsequent forms of care in general and specialized palliative care with particular consideration of emergency advance planning, if necessary
- If necessary, referral to qualified and continuous support services for relatives
- The time spent by doctors from the palliative care service, nursing staff from the palliative care service and representatives of the above-mentioned areas of the palliative care service on the patient and on the patient's relatives/caregivers is added up over the entire inpatient stay and coded accordingly. If two or more representatives from different professional groups of the treatment team are deployed simultaneously, the respective employee minutes are added up

Definition of other OPS-Codes

**1-510 Biopsy of intracranial tissue through incision and trepanation of skull bones**

**1-511 Stereotactic biopsy of intracranial tissue**

**5-015 Excision and destruction of diseased intracranial tissue**

**8-522 High-voltage radiotherapy**

**8-523 Other high-voltage radiotherapy (stereotactic and whole body radiation therapy)**

Radiotherapy includes regular documentation using suitable systems (film, portal imaging system). Each fraction must be coded individually. A fraction includes all settings and irradiation fields for irradiating a target volume. A target volume is the body volume that can be captured without patient repositioning or table shifting using appropriate field arrangements and irradiated with a fixed dose according to a specific dose-time pattern.

One or more simultaneously integrated boost irradiations must be coded separately (8-52e)

The irradiation simulation (8-528 ff.) and the irradiation planning (8-529 ff.) must be coded separately

**8-54 Cytostatic chemotherapy, immunotherapy and antiretroviral therapy**

Incl.: Therapy with immunomodulators or modified monoclonal antibodies

Info: Cytostatics, inhibitors, antibodies and supportive medications with their own OPS code in Chapter 6 Medications are coded with a code from 6-00 in addition to a code from 8-54 (examples: clofarabine, parenteral (6-003.j ff.), filgrastim, parenteral (6-002.1 ff.), rituximab (6-001.h ff.) (6-001.j ff.) alone or e.g. with R-DHAP)

The following information is only to be used for codes 8-542 ff., 8-543 ff. and 8-544:

Chemotherapy is administered according to the protocol-compliant duration of the subcutaneous injections administered during the inpatient stay. or intravenous chemotherapy. The daily specifications specified in the chemotherapy protocol are decisive for determining the duration of chemotherapy to be coded. Individually necessary delays are not taken into account. The days on which chemotherapy is administered count. For overnight administration, only the day on which administration was started counts. Breaks of a maximum of one day are only counted if they are a regular part of the respective chemotherapy protocol. Breaks of two days or more mean that a new code must be specified

The following substances can be counted: Alkylating substances

**8-542 Non-complex chemotherapy**

Info: These codes are to be used for subcutaneous or intravenous chemotherapy with 1-2 substances (cytostatics, inhibitors) as one-day chemotherapy

**8-543 Moderately complex and intensive block chemotherapy**

Info: Within the chemotherapy block, at least one substance (cytostatics, inhibitors) is administered subcutaneously or intravenously on at least 2 days or at least 3 substances as a one-day chemotherapy, or complex and intensive chemotherapy is administered with complex, measurement-dependent therapy control

**8-547 Immunotherapy**

8-547.0 With non-modified antibodies

Incl.: Therapy e.g. with rituximab in patients with lymphomas, alemtuzumab in patients with CLL, herceptin in patients with breast cancer, bevacizumab in patients with colorectal cancer

8-547.1 With modified antibodies

Incl.: Therapy with cytotoxins

Info: For conjugates of a monoclonal antibody and a cytotoxic substance, the cytotoxic substance is included in the code and is not to be counted additionally under 8-542 ff. to 8-544

8-547.2 With immunomodulators

Incl.: Therapy with interleukin 2, interferon or tumor necrosis factor alpha
